# Supplementary material for: Comparative genomics provides new insights into the diversity, physiology, and sexuality of the only industrially exploited tremellomycete: Phaffia rhodozyma
Source: BMC Genomics. 2016 Nov 9;17:901. doi: 10.1186/s12864-016-3244-7 (PMC5103461; doi:10.1186/s12864-016-3244-7)
Supplement: Additional file 6: — List of orphan genes with links to PFAM (related to Additional file 1: Table S1). (ZIP 1428 kb) [file 12864_2016_3244_MOESM6_ESM.zip › PFAM_UNIQUE/G04292_P.html]

#                                                                            --- full sequence --- -------------- this domain -------------   hmm coord   ali coord   env coord
  
# target name        accession   tlen query name           accession   qlen   E-value  score  bias   #  of  c-Evalue  i-Evalue  score  bias  from    to  from    to  from    to  acc description of target
  
#------------------- ---------- ----- -------------------- ---------- ----- --------- ------ ----- --- --- --------- --------- ------ ----- ----- ----- ----- ----- ----- ----- ---- ---------------------
  
G04292\_P             -            424 Bacteriocin\_IIc      PF10439.4     65       9.4    5.1   7.5   1   1     0.018        18    4.3   7.5    11    53   358   395   356   402 0.58 - Bacteriocin class II with double-glycine leader peptide  
G04292\_P             -            424 DSS1\_SEM1            PF05160.8     63      0.28    9.7   2.5   1   1   0.00021      0.63    8.6   2.5    46    61   346   361   334   363 0.90 - DSS1/SEM1 family  
G04292\_P             -            424 DUF605               PF04652.11   380       5.9    4.8  28.1   1   1      0.15         9    4.2  28.1   163   346   133   352    63   362 0.68 - Vta1 like  
G04292\_P             -            424 EAF                  PF09816.4    109   0.00012   20.7   0.0   1   1   5.6e-08   0.00034   19.2   0.0    14   105    48   155    27   159 0.68 - RNA polymerase II transcription elongation factor  
G04292\_P             -            424 TFIIA                PF03153.8    375    0.0061   15.2   5.8   1   1   0.00015     0.014   14.0   5.9   146   297   183   359   161   423 0.51 - Transcription factor IIA, alpha/beta subunit
